# Psychological Distress and Relational Intimacy following Sexual Violence: A Longitudinal Study

**DOI:** 10.5334/pb.1240

**Published:** 2024-12-17

**Authors:** Dagmar Stockman, Katarzyna Uzieblo, Elizaveta Fomenko, Heather Littleton, Ines Keygnaert, Gilbert Lemmens, Lesley Verhofstadt

**Affiliations:** 1 Department of Experimental Clinical and Health Psychology, Ghent University, Ghent, Belgium; 2 Expertise Network People and Society, Artevelde University College of Applied Sciences, Ghent, Belgium; 3 Faculty of Psychology and Educational Sciences Campus Kulak, Catholic University Leuven, Kortrijk, Belgium; 4 Department of Criminology, Vrije Universiteit Brussel, Brussels, Belgium; 5 Helpline 1712, Centre for General Well-being, Leuven, Belgium; 6 International Centre for Reproductive Health, Department of Public Health and Primary Care, Ghent University, Ghent, Belgium; 7 Lyda Hill Institute for Human Resilience, University of Colorado, Colorado Springs, United States; 8 Department of Psychiatry, Ghent University Hospital, Ghent, Belgium; 9 Department Head and Skin –Psychiatry, Ghent University, Ghent, Belgium

**Keywords:** Sexual violence, posttraumatic stress, relationship functioning, sexual intimacy, emotional intimacy

## Abstract

Increasing evidence shows how distress following sexual violence might spill over to victims’ romantic relationship functioning. However, studies investigating the reverse spillover between relationship functioning and psychological distress following sexual violence are lacking. The current study therefore aimed to investigate the bidirectional association between victims’ psychological distress (posttraumatic stress, depression, anxiety and stress) and emotional and sexual intimacy using a three-wave yearly survey study of a community sample of sexual violence victims (*N* = 274, 89% women, 3% men and 8% trans persons, *M_age_* = 32 years, *SD* = 10.7). Results show evidence for spillover effects for posttraumatic stress and stress on emotional intimacy and for anxiety on sexual intimacy. In addition, reverse spillover effects for emotional intimacy were found on all indicators of psychological distress and for sexual intimacy on depression and anxiety. These results suggest that victims’ individual and relational functioning is bidirectionally associated. In addition, results suggest that targeting intimacy levels within romantic relationships might improve victims’ individual functioning and vice versa. Future research that includes dyadic assessment could enhance our understanding of the bidirectional associations between individual functioning and couple functioning following sexual violence.

Sexual violence is defined as any sexual act against someone’s will, committed by any person regardless of their relationship to the victim, in any setting (WHO, 2015, p.4). Having experienced sexual violence in adolescence or adulthood is associated with many negative mental health outcomes, including posttraumatic stress symptoms as well as anxiety and mood disorder symptoms (Dworkin et al., 2017; Stockman et al., 2023). Increasing evidence suggests that victims of sexual violence frequently experience difficulties in their ongoing and future romantic relationships, with research supporting that experiencing negative psychological distress likewise negatively affects victims’ relationship functioning (DiMauro & Renshaw 2019; Georgia et al, 2018; Vitek & Yeater, 2021). Meanwhile, studies among individuals experiencing other forms of stress and adversity have shown that individuals’ relationship functioning (e.g., relationship satisfaction, intimacy, partner warmth and partner hostility) also affects their individual functioning, including their levels of depression and posttraumatic growth (Barr et al., 2013; Canavarro et al., 2015). As such, experiencing relationship difficulties might also negatively affect victims’ mental health, whereas relationship adjustment might have a buffering effect against mental health issues. Until present, there is no research providing clarity about any potential bidirectional association between relationship functioning and psychological distress in victims of sexual violence. The aim of the current study therefore is to investigate the bidirectional association between victims’ mental health functioning and their relationship functioning. In what follows, available theoretical and empirical evidence for this association will be elaborated upon by extrapolating from the existing literature on relationship functioning and psychological distress in victims of other trauma types.

## Stress-spillover: Relational Effects of Posttraumatic Stress

Spillover means that affective states that originate from one life domain carry over to other life domains (Lourel et al., 2009). In the case of affective states stemming from a negative stressor, this process is referred to as negative spillover or ‘stress spillover’ (Bolger et al., 1989). Applied to intimate relationships, distress stemming from a stressor external to the intimate relationship (e.g., a work-related stressor) spills over to the relationship. A multitude of studies document the spill-over effect of stressors from multiple domains (e.g., work-related, daily, health-related, exam-related stressors) to intimate relationships (Casas & Benuto, 2022; Cooper et al., 2019; Harper et al., 2000; Totenhagen et al., 2017; Tracy et al., 2021; Tuttle et al., 2018). In line with these findings, it seems probable that experiencing sexual violence would likewise affect victims’ intimate relationship(s). Supporting this possibility, cross-sectional studies among samples of students, veterans, refugees, and community samples find that individuals with a sexual assault history report worse relationship functioning as indicated by more negative interactions, more relationship distress, less relationship satisfaction and lower overall emotional/sexual intimacy levels than those without an assault history (Collibee & Furman, 2014; Flanagan & Furman, 2000; Georgia et al., 2018; Rizkalla & Segal, 2019; Rothman et al., 2021; Stahlman et al., 2015). Notably, in a prospective study conducted over the course of eight years among college students, Collibee and Furnam (2014) found sexual assault victims reported an increase in negative interactions with their intimate partner immediately following the assault. Further, these negative couple interactions persisted through the yearly follow-up assessment.

According to the cognitive-behavioral interpersonal theory of posttraumatic stress (Monson et al., 2010), a traumatic event -like sexual violence- can affect a person’s ability to connect with their romantic partner due to their experience of emotional, cognitive and behavioral posttraumatic stress symptoms. For instance, victims experiencing increased vigilance for possible danger cues might also be vigilant toward signs of untrustworthiness in their partner. In addition, avoidant behavior and emotional numbing can contribute to emotional and physical withdrawal from one’s partner, whereas hyperarousal might strengthen feelings of irritability towards the partner. These symptoms might also affect the victim’s need for and perceptions of intimacy in their relationship(s). The partner, in turn, might interpret these behaviors as being intentionally directed towards them rather than an expression of the victim’s individual distress. As such, this might decrease their supportive behavior which affects the relationship even further. Evidence in line with this theory is found among individuals reporting psychological distress following a diversity of traumatic events. For instance, cross-sectional studies among former war-prisoners and refugees showed that those who met the criteria for posttraumatic stress disorder (PTSD) reported more relationship difficulties (e.g., increased marital distress, decreases in relationship satisfaction, intimacy and constructive communication) than those without a PTSD diagnosis (Cook et al., 2004; Rizkalla & Segal, 2019). Similarly, an observational study among couples found that partners with elevated posttraumatic stress levels following a stressful life event experienced more fear whenever their partner expressed intimate behavior (e.g., caring, validation, understanding) than those without elevated posttraumatic stress (Leifker et al., 2015). Another cross-sectional study among motor vehicle accident victims showed that depressive symptoms, but not posttraumatic stress symptoms, were associated with worse relationship functioning (Beck et al., 2009).

For sexual violence victims, similar results are found. For example, a study of couples found that sexual assault victims psychological distress was negatively associated with both partners’ relationship satisfaction levels (Georgia et al, 2018). In addition, a study conducted by DiMauro and Renshaw (2019) demonstrates how posttraumatic stress symptoms are associated with decreased constructive communication and sexual satisfaction in victims.

However, longitudinal studies provide a less consistent picture of the association of posttraumatic stress symptoms with relationship functioning. In military and veteran samples, more posttraumatic stress was predictive of worse relationship adjustment over time (Creech et al., 2019; Erbes et al., 2012; Levin et al., 2017). In contrast, in a study of motor vehicle accident victims, posttraumatic stress did not significantly predict future relationship dissatisfaction (LeBlanc et al., 2016). However, no studies to our knowledge have examined the longitudinal association between posttraumatic stress symptoms and relationship functioning in sexual violence victims.

## The Effect of Relationship Adjustment on Psychological Distress

Most work on spillover effects in the trauma and romantic relationship literature focuses on how individual distress stemming from external stressors affects romantic relationships. However, in the past, scholars have argued that there is also a ‘reverse spillover effect’. Romantic relationships are deemed both an important source of distress and a vital support source for individuals (Beach et al., 1993; Simon & Barrett, 2010). In the case of a traumatic experience, it is assumed that higher romantic relationship quality will lead to better adjustment in the trauma victim over time.

There are some cross-sectional studies that explicitly take this reverse spillover perspective, theorizing that better intimate relationship functioning might indeed be a protective factor against trauma victims’ psychological distress. A study from Fredman and colleagues (2010) among women who were exposed to a serious flooding disaster found that better relationship adjustment was associated with less posttraumatic stress. Additionally, in two cross-sectional studies, relationship satisfaction proved to be a protective factor for hazardous drinking behavior in response to posttraumatic stress symptomatology in female veterans (Blais et al., 2021), and likewise relationship adjustment buffered against suicidal ideation in former prisoners of war (Zerach et al., 2019). Notably, a longitudinal study among Yom Kippur war veterans not only found posttraumatic stress to be predictive of worse marital adjustment, but also found marital adjustment to be a protective factor against future posttraumatic stress (Levin et al., 2017). Furthermore, among motor vehicle accident victims, relationship satisfaction significantly predicted a subsequent decrease in some posttraumatic stress symptoms, such as re-experiencing, emotional numbness, and irritability (Leblanc et al., 2016). However, in a sample of 9/11 war veterans, no evidence was found for similar reverse spillover effects (Creech et al., 2019). Cross-sectional or longitudinal studies taking the perspective of how intimate relationship functioning might affect sexual violence victims’ mental health are – to the best of our knowledge – non-existent.

## The Current Study

Existing research has found some evidence for bidirectional spillover effects between distress following several types of traumatic events (e.g., combat exposure, motor vehicle accident) and relationship functioning. The existing research is, however limited in two respects: (1) there is a lack of longitudinal research on the intimate relationships of sexual assault victims, (2) there is a lack of research adopting a bidirectional perspective when studying (longitudinal) associations between victims’ psychological distress and their intimate relationship experiences. The current study therefore sought to examine whether sexual violence victims’ psychological distress was longitudinally and bidirectionally associated with victims’ romantic relationship functioning.

The current study examined multiple indicators of psychological distress following sexual violence. Specifically, alongside posttraumatic stress symptoms, symptoms of depression, anxiety and stress were assessed because of the following reasons: (1) the comorbidity between posttraumatic stress disorder and other mental health issues is believed to be high (Sareen, 2014), (2) victims are frequently suffering from depression, anxiety and other stressor-related conditions (Dworkin et al., 2017) and (3) previous research among couples has shown that depression, anxiety and stress can have significant negative effects on romantic relationships (Porter & Chambless, 2017; Randall & Bodenmann, 2009; Sharabi et al., 2015). In addition, romantic relationship functioning was operationalized as victims’ emotional and sexual intimacy levels. Emotional intimacy refers to experiencing an emotional closeness between partners; feeling able to share emotions with the partner and feeling understood by the partner. Sexual intimacy entails sharing sexual experiences and addressing both partners’ sexual needs (Schaefer & Olson, 1981). The rationale for choosing emotional and sexual intimacy as indicators for romantic relationship functioning is threefold: (1) lack of intimacy is found to be a common motive for relationship counseling/therapy (Doss et al., 2004), (2) the process of obtaining emotional intimacy might aid the couple in coping with the assault impact together (Bodenmann, 2005; López-Zerón & Blow, 2015) and (3) when considering psychological distress following sexual violence and its potential impact on intimate relationships, emotional and sexual intimacy seem to be the intimacy forms that might be most affected (Schaefer & Olson, 1981). We anticipated that feelings of distrust, avoidant behavior, emotional numbing, and increased irritability following sexual violence might specifically disturb emotional and sexual intimacy processes between partners (Mills & Turnbull, 2001; 2004).

In line with the theoretical and empirical evidence in victims who experienced other interpersonal and non-interpersonal traumatic events we specifically hypothesized that:

1. Higher levels of psychological distress (posttraumatic stress, depression, anxiety and stress) in victims of sexual violence would predict a future decrease in their reported emotional and sexual intimacy.
2. Higher levels of emotional and sexual intimacy would predict a future decrease in sexual violence victims’ psychological distress.

## Method

### Participants

A Flanders (i.e., the Dutch speaking region in Belgium) community sample of 274 sexual assault victims (243 women, 9 men and 22 trans persons, see Table 1) that were in a romantic relationship at the time of participation filled out at least one wave of the survey as part of a study about the psychosocial impact of sexual violence. In order to increase the sample size, the entire sample consisted of two cohorts: one cohort who completed three yearly survey waves starting in 2019–2020 and one cohort who completed two yearly survey waves starting in 2020–2021. Victims who indicated having experienced sexual violence within their current romantic relationship were excluded. The mean age of participants at enrollment was 32 years (*SD* = 10.7, *range* = 18–65 years). Of the total sample, 6.9% (*n* = 19) had completed middle school, 30.2% (*n* = 83) had completed high school, and 62.7% (*n* = 172) had obtained an undergraduate or graduate degree from a college/university. Regarding employment status at enrollment, 55.8% (*n* = 153) were employed, 1.8% (*n* = 5) were looking for work, 30.2% (*n* = 83) were students, 1.1% (*n* = 3) were retired and 10.9% (*n* = 30) were unable to work at the time of the participation. Sixty-six point one per cent (*n* = 181) identified as heterosexual, 33.9% (*n* = 93) identified as lesbian, gay or bisexual (LGB). Participants were in their current intimate relationships at enrollment for an average of 74 months (*SD* = 77.9, *range* = 3–416 months). Regarding relationship status at enrollment, 39.1% (*n* = 107) reported not being married/living together, 59.9% (*n* = 164) reported living together/being married.

**Table 1:** Descriptive statistics of psychological distress and intimacy per wave.


OUTCOME VARIABLE	WAVE	*N*	MEDIAN	MEAN	SD

**Depression**	1	221	6.00	7.33	5.56

	2	69	5.50	6.73	5.35

	3	51	6.00	6.08	4.81

**Anxiety**	1	221	5.00	6.77	5.46

	2	69	4.50	5.71	4.84

	3	51	4.50	5.26	4.17

**Stress**	1	221	9.00	9.35	4.93

	2	69	7.50	8.68	5.01

	3	51	8.50	8.00	4.73

**Posttraumatic stress**	1	221	4.00	4.07	2.20

	2	69	2.00	2.28	2.16

	3	51	2.00	2.34	2.26

**Emotional intimacy**	1	221	22.00	21.96	5.53

	2	69	24.00	23.38	4.55

	3	51	22.50	22.04	5.87

**Sexual intimacy**	1	221	23.00	21.86	5.53

	2	69	24.00	23.41	4.78

	3	51	23.00	22.34	4.77


*Note*. SD = Standard deviation.

### Procedure

The current study was part of a series of qualitative and quantitative studies in the context of a PhD project. The study was carried out in line with the World Health Organization’s (WHO, 2007) recommendations on conducting research with victims of sexual violence and was approved by the Committee for Medical Ethics of Ghent University Hospital (B67201940809). Similar to previous research with sexual assault victims (see e.g., Campbell et al., 2004), an adaptive sampling method (Thompson & Seber, 1996) was used. A call for participation was distributed in multiple regions across Flanders and in a wide range of clinical (e.g., hospitals, sexual assault referral centers, private practices of physicians and psychologists, counseling centers) and non-clinical settings (e.g., fitness centers, public library, bakery, hair salons, bars, social media) using paper and digital versions of posters and flyers. These posters and flyers introduced the study as being on the psychosocial impact of sexual violence that occurred after the age of 16 (i.e., the legal age of consent at the time of the study in Belgium). Alongside our inclusion criteria (individuals 18 years or older having experienced one or more instances of sexual violence after the age of 16), the definition of sexual violence (i.e., any sexual act against someone’s will; WHO, 2015, p.4) and the survey link was provided. It was stated that participation was voluntary and would be handled confidentially. In addition, contact details of the researcher, professional support sources, and a website link with more detailed information about the study were provided. No compensation was provided for study participation.

The survey was developed using Qualtrics. After clicking on the survey link, participants were directed to the informed consent form, which had to be filled out completely to proceed to the survey. The survey consisted of a set of questionnaires assessing sexual violence experiences, psychological distress, and relationship outcomes. Those measures unrelated to the sexual violence event (i.e., depression, anxiety, stress, intimacy) were administered in a first part of the survey in order not to influence participants’ answers because of distressing emotions evoked by the questionnaires on sexual violence and posttraumatic stress. Contact information of support sources were stated on every survey page. At the end of the survey, participants were able to indicate if they would like to be invited to complete future study surveys. Those who answered affirmatively were asked to generate a self-identification code based on their answers on several personal questions (e.g., number of older siblings, second letter of father’s name) and were informed that these questions would be used to link their answers across surveys. Subsequently, they were redirected to a separate Qualtrics survey where they could leave their e-mail address.

The study was conducted in three waves. Data-collection for the first wave was initiated in November 2019 and finalized by the start of the corona-virus 2019 lockdown (i.e., March 2020) as the authors thought the lockdown might affect survivors’ answers to the included measures. The subsequent two waves followed the same time-frame. An overview of measurements administered for each cohort and each wave can be found in Supplementary Table A.

### Measures

#### Sociodemographics

In the beginning of the survey, victims were asked to report their birth year, their sex assigned at birth (male, female, intersex), their gender identity (assessed using two dimensional scales on which they were asked to indicate to what extent they identify as male or female), their sexual orientation (whether they were romantically attracted to men, women, both or neither), nationality, (Belgian or other), highest degree obtained, employment status, living situation (e.g., whether they were married or cohabitating) and relationship duration.

#### Adult Sexual Violence

Whether respondents experienced sexual violence after the age of 16 and in the past 12 months was assessed using the original Dutch version of the ‘Understanding the Mechanisms, Nature, Magnitude and Impact of Sexual violence’ (UNMENAMAIS) questionnaire. The questionnaire is based on existing validated questionnaires (see Keynaert et al., 2021 for an overview of the survey development) and consists of 17 behaviorally specific questions assessing hands-off (8 items, e.g., “Someone stared at me in a sexual way or looked at my intimate body parts [e.g., breasts, vagina, penis, anus] when I didn’t want it to happen.”; “Someone distributed naked pictures or videos of me directly or over the internet [including email, social networks and chat platforms] when I didn’t want it to happen”) and hands-on (nine items, e.g., “Someone removed [some of] my clothes against my will”; “Someone tried, but did not succeed, in putting their penis, finger(s) or object(s) into my vagina or anus against my will”) sexual violence.

#### Child Abuse History

Two items from the Trauma History Screen (Carlson et al., 2011) were used to assess whether victims had any experiences of physical (‘Have you ever been hit or kicked hard enough to injure – as a child?’) and sexual child abuse (‘Have you ever been forced or made to have sexual contact – as a child?’) with a dichotomous response option (yes/no). Support for the entire questionnaire’s psychometric properties (i.e., one-week and two-month test re-test reliability and convergent validity) was found in community, clinical, veteran and college samples (Carlson et al., 2011). The scale was translated into Dutch using multiple (i.e., multiple translators) and backward translation (i.e., translating the items back to English and comparing the backward translated version to the original version), using the guidelines of the International Test Commission (2017).

#### Help-seeking Behavior

Respondents were asked to whom they had disclosed their experiences with sexual violence. To respond to this question, respondents had to indicate with ‘yes (1)/no (0)’ whether they had disclosed to a wide range of informal (e.g., partner, family, friends) and formal support sources (e.g., hospital, psychiatrist, sexual assault care center). For the current study, two dichotomous variables were formed. The first indicated whether the victim disclosed to their current romantic partner whereas the second indicated whether victims disclosed to one or more of the listed formal support sources.

#### Depression, Anxiety and Stress Symptoms

Depression, anxiety and stress symptoms were assessed with the 21 item Dutch version of the Depression, Anxiety and Stress Scale (DASS-21, Lovibond & Lovibond, 1996; Dutch version, De Beurs, 2001). The DASS-21 consists of three 7-item scales assessing participants’ distress in the past week (e.g., depression: “I felt I wasn’t worth much as a person”, “felt that I had nothing to look forward to”; anxiety: “I was aware of the action of my heart in the absence of physical exertion [e.g., sense of heart rate increase, heart missing a beat]”, I felt scared without any good reason”; stress: “I was intolerant of anything that kept me from getting on with what I was doing”, “I felt that I was rather touchy”). Responses were given on a four-point rating scale ranging from ‘Did not apply to me at all (0)’ to ‘Applied to me very much or most of the time (3)’. In order to be able to interpret the scale scores in a similar way as the other scales, items were summed per set of scales leading to three scales ranging from zero to 21 to yield a total depression, anxiety and stress score. Previous studies have found support for the scale’s factor structure, internal consistency and convergent validity in clinical and community samples (Antony et al., 1998). For the current study, Cronbach’s alphas for all waves combined were respectively .91, .88 and .90.

#### Posttraumatic Stress Symptoms

Posttraumatic stress symptoms were assessed using the 7-item Short Screening Scale for DSM-IV posttraumatic stress disorder (PTSD screener; Breslau et al., 1999), which explored the presence of emotional numbing, avoidance, and arousal symptoms in the month before completion of the questionnaire (e.g., “making efforts to avoid activities, places, people that arouse recollections of the trauma, experiencing an exaggerated startle response”). The scale was translated into Dutch using multiple and backward translation (International Test Commission, 2017). It has a response format of ‘yes (1)/no (0)’ with scores summed (possible range 0–7). A score of four is regarded as an indication of probable PTSD, with this cutoff score having a sensitivity of 80% and a specificity of 97% in a community sample (Breslau et al., 1999). In addition, support for the scale’s one month test re-test reliability was found in a treatment-seeking veteran sample (Kimerling et al., 2006). A test re-test reliability analysis was not conducted for the current study because of the large time-period between both waves.

#### Emotional and Sexual Intimacy

Two subscales of the Personal Assessment of Intimacy in Relationships (PAIR; Schaefer & Olson, 1981) were administered to assess emotional and sexual intimacy. Specifically, the subscales emotional intimacy (e.g., “My partner listens to me when I need someone to talk to”, “I often feel distant from my partner.”) and sexual intimacy (e.g., “Sexual expression is an essential part of our relationship”, “I am satisfied with our sex life”) were used. The PAIR was translated into Dutch using multiple and backward translation (International Test Commission, 2017). Each scale contained six statements about participants’ current intimacy experiences rated on a 5-point rating scale ranging from ‘Does not apply to me/my relationship at all (1)’ to ‘Applied to me very much or most of the time (5)’. Item scores per scale were summed resulting in total scores ranging from 6 (low levels of intimacy) to 30 (high levels of intimacy). Evidence for the English PAIR’s factor structure, internal consistency and convergent and discriminant validity has been found in a sample of married community couples and ex-prisoners of war (Cook et al., 2004; Schaefer and Olson, 1981). The combined Cronbach’s alpha for all waves in the current study for emotional and sexual intimacy were respectively .89 and .83.

### Data-cleaning and Data-analysis

For the first cohort starting in November 2019, the online survey was started by 831 respondents in the first wave with 118 respondents indicating they wanted to participate in future waves and left a code, 125 started in the second wave and 133 in the third wave. In the second cohort (starting in November 2020), 778 respondents started the online survey in the first wave with 108 respondents indicating they wanted to participate in future waves and left a code whereas 134 started in the second wave. Of the 2001 responses across surveys, 27.8% (*n* = 558) were excluded because the respondent did not give informed consent, 5.0% (*n* = 101) because the participant did not complete all demographic questions in wave 1, 1.9% (*n* = 39) were duplicate responses, and 10.9% (*n* = 219) did not meet the inclusion criteria. Additionally, for the current study, 1.7% (*n* = 34) of responses were excluded because they experienced sexual intimate partner violence by their current partner, 22.6% were not in a relationship at the time of participation (*n* = 453) and 1.5% (*n* = 31) were in their current relationship for less than three months. Another 9.5% (*n* = 191) of responses were eliminated for not including an identification code to link responses and 1.8% (*n* = 36) for having missing values on one of the key outcome variables for this study leaving us with a final sample of 274 unique participants. Out of the 274 unique participants in the study, a total of 339 observations were collected. Notably, 22.9% (*n* = 63) of the participants contributed data at multiple measurement points, allowing for a longitudinal perspective on their experiences. Eighty-one percent (*n* = 222) of the respondents completed all relevant scales for the current study during wave 1, 25.2% (*n* = 69) during wave 2 and 17.8% (*n* = 49) during wave 3.

Data was imported into SPSS27 for initial data cleaning and data manipulation. All statistical analyses were carried out by the third author and were conducted with R software version 4.0.3. Dropout analyses were carried out by calculating correlations between having participated in multiple waves and our variables of interest. To test our first hypothesis on the longitudinal association of psychological distress and intimacy, we employed generalized least squares (GLS) random-effects modelling (Fitzmaurice et al., 2004; Heagerty et al., 2002; Verbeke & Molenberghs, 2000). Specifically, we tested two models: one with emotional intimacy as our dependent variable and one with sexual intimacy as our dependent variable. We included posttraumatic stress, depression, anxiety and stress symptoms as independent variables. Potential covariates included in the model were cohort number, socio-demographic variables, having experienced sexual and/or physical abuse as a child, whether respondents disclosed whether they have been exposed to sexual violence to a formal support provider and whether they disclosed to their partner. Wave number (time) was included as a control variable to account for time-related effects and was treated as a fixed effect. The longitudinal regression models included a random effect for respondent identifier, adjusting for the potential confounding variables. Multicollinearity between all predictors was assessed using variance inflation factor (VIF); with no values exceeding the standard threshold of 10 (Myers, 1990). Profile plots, scatter plot smoothers, box plots and scatter plot matrices were used to gain a first insight in the potential model choice for the mean and the covariance. We checked whether a model with only randomly varying intercepts is defensible by refitting the model without random slopes using Restricted Maximum Likelihood (REML) estimation. The results were significant (p < 0.001), suggesting strong evidence for random slopes. Before choosing the right covariance pattern, the different covariances between the groups were explored. To select the right covariance, we made use of the Likelihood ratio test to compare all covariance models with the unstructured model. All covariance models were rejected and thus the unstructured covariance pattern was selected. In a next step we started model building with a stepwise backward approach. The nested conditional mean models were compared using the likelihood ratio test. In a first step we added all variables of interest, as well as an interacting effect of time on all of these variables. In a second step we removed the interacting effects as they were all found to be non-significant (*p* < 0.05). All used variables were kept in all models, allowing for the four final models to be compared to each other.

Finally, four additional GLS models, with the same approach as mentioned above, were used to test the second hypothesis: the longitudinal association between intimacy and psychological distress. For each model one of the indicators of psychological well-being (i.e., depression, anxiety, stress and posttraumatic stress symptoms) was added as a dependent variable whereas emotional and sexual intimacy were added as independent variables. Wave number (time) was included as a control variable to account for time-related effects. The longitudinal logistic regressions with binary response, included a random effect for patient identifier. Multicollinearity between all predictors was assessed using variance inflation factor (VIF); with no values exceeding the standard threshold of 10. All main effects were included in the model.

Our longitudinal design with three waves and the inclusion of wave number (time) as a variable allowed us to measure the bidirectional associations between psychological distress and intimacy, ensuring a robust assessment of their temporal relationships.

## Results

### Descriptive Analysis

Almost half (46.7%; *n = 128*) of the respondents reported a history of child abuse (physical or sexual). All participants reported an experience of hands-off (98.9%; *n = 271*) and/or hands-on (97.4%; *n = 267*) sexual violence in their life. Most respondents disclosed their experience of sexual violence to their partner, whereas half disclosed to a counsellor, psychologist or psychiatrist (50.0%; *n = 137*).

Table 1 shows the included scales’ medians, means and standard deviations. Compared to the DASS norms (Lovibond & Lovibond, 1996), half of the participants scored normal to moderate on the depression, anxiety and stress scales whereas the other half scored moderately to extremely severe on all waves. During the first wave half of the participants scored higher than the four-point cut-off indicating an increased likelihood for post-traumatic stress disorder (Breslau et al., 1999). This number decreased during the subsequent waves as only half of all participants scored higher than two. The sexual and emotional intimacy scores from our sample were similar to general intimacy scores among ex-prisoners with a PTSD diagnosis (Cook et al., 2004).

Correlation analysis showed that all indicators of psychological distress were significantly negatively associated with both sexual and emotional intimacy (–.13 < *r* < –.27, *p* < .001), except for an insignificant association between anxiety, stress and sexual intimacy (see Supplementary Table B). In addition, drop-out analyses showed that higher stress levels and lower emotional and sexual intimacy levels during the first wave were associated with a higher likelihood of not participating during the following waves (*r_stress_* = –.10, *p* < .05; *r_emotional intimacy_* = .11, *p* < .001; *r_sexual intimacy_* = .15, *p* < .001).

### Hypothesis 1: Victims’ Psychological Distress Predicts a Decrease in Intimacy

Table 2 shows the results of the generalized linear models for emotional and sexual intimacy. With regard to control variables, the age of the respondents and the duration of their relationship were found to significantly improve the model for both intimacy types. Older participants scored lower on sexual intimacy and participants who have been together with their partner for a longer period of time reported lower scores on both emotional and sexual intimacy. No significant effects were found for wave, cohort, sexual orientation, education, child abuse history, sexual violence in the past 12 months and disclosure of the assault experience to the partner or a professional. These were therefore not included in the final Table (see Supplementary Table C for a detailed overview).

**Table 2:** Solution for fixed effects of the final models for emotional and sexual intimacy.


	EMOTIONAL INTIMACY				SEXUAL INTIMACY			

EFFECT	ESTIMATE	95% C.I. (WALD)		P (LRT)	ESTIMATE	95% C.I. (WALD)		P (LRT)
	
**Age**	–0.08	–0.15	–0.01	**<.001**	**–0.12**	**–0.20**	**–0.05**	**<.001**

**Gender** (ref. Male)				.867				**.005**

Female	0.65	–2.26	3.56		0.46	–2.25	3.18	

Transgender	0.65	–2.80	4.09		–2.41	–5.64	0.83	

**Relationship duration (in years)**	**–0.26**	**–0.37**	**–0.14**	**<.001**	**–0.19**	**–0.30**	**–0.08**	**<.001**

**Employment** (ref. Working)				**.034**				.106

Student	–0.51	–1.92	0.91		–0.15	–1.52	1.22	

(Temporarily) not working	–1.27	–2.89	0.34		–0.65	–2.20	0.90	

**Depression**	–0.04	–0.20	0.11	**<.001**	–0.05	–0.20	0.10	**<.001**

**Anxiety**	–0.02	–0.19	0.15	.074	**–0.20**	**–0.36**	**–0.03**	**.003**

**Stress**	**–0.20**	**–0.38**	**–0.02**	**.046**	–0.03	–0.20	0.14	.794

**Posttraumatic stress**	**–0.41**	**–0.70**	**–0.13**	**.004**	–0.13	–0.40	0.15	.365


*Note*. C.I.: Confidence Interval; LRT = Likelihood Ratio Test; LGB+ = self-identified as lesbian, gay, bisexual, pan-/omnisexual, asexual, or other; SV = Sexual Violence.

Generally, in line with hypothesis 1, psychological distress was negatively related to intimacy. However, not every indicator of psychological distress was associated with emotional and/or sexual intimacy. Specifically, respondents who reported higher levels of stress and posttraumatic stress symptoms during earlier waves, experienced lower levels of emotional intimacy in later waves. Respondents with higher levels of anxiety symptoms experienced subsequent lower sexual intimacy. Although depression symptoms significantly improved the model for both emotional and sexual intimacy, we cannot interpret its parameter estimates as zero falls within the 95% confidence interval. This means that one unit increase in depression is statistically too small to affect emotional or sexual intimacy.

### Hypothesis 2: Intimacy Predicts a Decrease in Victims’ Psychological Distress

Table 3 shows the results of the generalized least squares models for the different psychological distress outcomes. Wave and disclosure to a formal support provider was found to improve all models significantly except the depression model, but only the parameter estimate of wave in the posttraumatic stress model can be interpreted since the 95% confidence interval for wave in this model does not contain zero. Specifically, posttraumatic stress symptoms decreased over subsequent waves. In addition, sexual orientation was found to be a significant predictor of psychological distress, with LGB+ participants having a higher risk of depression, anxiety and stress symptoms compared to heterosexual participants. Additionally, unemployed participants showed an increased risk of depression compared to employed participants. A final significant control variable was having experienced child abuse. Participants who reported a history of child abuse reported higher levels of depression, anxiety, stress, and posttraumatic stress symptoms. No significant effect was found for cohort, gender, the occurrence of sexual violence in the past 12 months and disclosure of a sexual violence experience to their partner. Therefore, these effects were left out in Table 3 and can be found in Supplementary Table D.

**Table 3:** Solution for fixed effects of the final models for depression, anxiety, stress, and posttraumatic stress.


	DEPRESSION				ANXIETY				STRESS				POSTTRAUMATIC STRESS			

EFFECT	ESTIMATE	95% C.I. (WALD)		P (LRT)	ESTIMATE	95% C.I. (WALD)		P (LRT)	ESTIMATE	95% C.I. (WALD)		P (LRT)	ESTIMATE	95% C.I. (WALD)		P (LRT)
			
**Wave (Time)**	–0.46	–1.24	0.32	.065	–0.46	–1.06	0.15	**.009**	–0.38	–1.03	0.27	**.012**	**–0.96**	**–1.30**	**–0.61**	**<.001**

**Age**	–0.12	–0.19	–0.04	.482	**–0.14**	**–0.22**	**–0.07**	**.005**	**–0.12**	**–0.19**	**–0.05**	**.004**	0.01	–0.02	0.04	**.030**

**Sexual orientation** (ref. Heterosexual)				**<.001**				**.001**				**<.001**				.133

LGB+	**2.00**	**0.83**	**3.16**		**1.44**	**0.29**	**2.58**		**1.45**	**0.35**	**2.55**		0.13	–0.34	0.60	

**Relationship duration (in years)**	0.08	–0.04	0.19	**<.001**	–0.05	–0.16	0.06	.187	–0.06	–0.16	0.05	.203	–0.03	–0.08	0.01	.330

**Education** (ref. No higher)				.465				**.003**				.121				.250

Higher	0.38	–0.73	1.49		–0.84	–1.94	0.26		–0.08	–1.13	0.97		0.10	–0.35	0.55	

**Employment** (ref. Working)				**<.001**				**<.001**				**.004**				**.001**

Student	0.72	–0.70	2.15		–0.36	–1.71	0.98		–0.37	–1.68	0.95		0.33	–0.24	0.91	

(Temporarily) not working	**2.65**	**1.05**	**4.25**		**1.83**	**0.31**	**3.35**		1.17	–0.32	2.66		0.40	–0.26	1.05	

**Child abuse** (ref. No)				**<.001**				**<.001**				**<.001**				**<.001**

Yes	**2.16**	**1.07**	**3.26**		**2.30**	**1.18**	**3.42**		**2.24**	**1.18**	**3.30**		**1.07**	**0.62**	**1.51**	

**Disclosure of SV to formal support provider** (ref. No)				.071				**.024**				**.017**				**<.001**

Yes	1.01	–0.05	2.07		**1.18**	**0.12**	**2.25**		**1.31**	**0.29**	**2.32**		**1.47**	**1.04**	**1.90**	

**Emotional intimacy**	**–0.18**	**–0.30**	**–0.06**	**<.001**	**–0.15**	**–0.26**	**–0.04**	**<.001**	**–0.21**	**–0.32**	**–0.10**	**<.001**	**–0.09**	**–0.14**	**–0.04**	**<.001**

**Sexual intimacy**	**–0.13**	**–0.26**	**–0.01**	**.040**	**–0.16**	**–0.28**	**–0.04**	**.007**	–0.09	–0.21	0.03	.129	–0.01	–0.06	0.04	.585


*Note*. C.I.: Confidence Interval; LRT = Likelihood Ratio Test; LGB+ = self-identified as lesbian, gay, bisexual, pan-/omnisexual, asexual, or other; SV = Sexual Violence.

In line with our second hypothesis, emotional intimacy was found to be a strong and significant predictor of all forms of psychological distress. Specifically, participants who reported higher levels of emotional intimacy in their relationship during an earlier wave showed lower levels of depression, anxiety, stress, and posttraumatic stress symptoms in later waves. Additionally, sexual intimacy was associated with lower levels of depression and anxiety. Victims who reported a higher level of sexual intimacy in their relationship, experienced lower future depression and anxiety symptoms.

## Discussion

The current study sought to examine whether sexual violence victims’ psychological distress (depression, anxiety, stress, and posttraumatic stress) was longitudinally and bidirectionally associated with victims’ emotional and sexual intimacy in their romantic relationship. Generally, our hypotheses were confirmed and as such two main conclusions can be drawn from our research results.

Our first main conclusion is that there is evidence for a *stress spillover effect* of individual distress in victims of sexual violence on emotional and sexual intimacy. Our results supported that higher posttraumatic stress and stress-symptoms in sexual violence victims predicted lower future emotional intimacy within romantic relationships. These findings are in accordance with the cognitive behavioral interpersonal theory of posttraumatic stress (Monson et al., 2010). Posttraumatic stress symptoms and stress symptoms might restrict the way in which victims are able to express themselves and their needs which potentially makes it difficult for a partner to understand them. This might contribute to the development of maladaptive relationship patterns such as demand-withdraw behavior (Cook et al., 2004; Eldridge & Christensen, 2002). Although specific symptoms were not assessed in the current study, symptoms of numbness and avoidance might be considered by the partner as emotional withdrawal and as such are responded to by the partner by either withdrawal behavior or more demanding behavior because the partner feels their own needs are not met. In turn, victims might not feel supported or understood or feel as if the partner does not understand their emotional intimacy needs. Conversely, stress, irritability, and arousal symptoms might be interpreted by the partner as demanding behavior which then is responded to by withdrawal. As such, again, the victim might be left feeling unsupported and misunderstood by their partner which contributes to a lower emotional intimacy in the relationship.

In a similar manner, victims’ anxiety levels are related to later decreases in their sexual intimacy ratings. However, unexpectedly, posttraumatic stress and stress symptoms were not associated with sexual intimacy. This finding, especially regarding posttraumatic stress symptoms, seems counterintuitive as research shows how sexual interactions might function as a trigger for posttraumatic symptoms among sexual violence victims (Orlando & Koss, 1983) and that victims’ psychological distress is associated with victims’ sexual functioning. One would therefore expect that posttraumatic stress symptoms and stress would affect sexual intimacy within their relationship. Possibly, our measurement of posttraumatic stress was not elaborated enough to capture this association. It is likely that some specific posttraumatic stress symptoms are more strongly associated with sexual intimacy in comparison to other symptoms. Previous research has shown, for instance, that emotional numbing seems to be an important predictor of decreased sexual functioning in veterans (Nunnink et al., 2010). It is possible that emotional numbing symptoms hamper the ability to experience sharing physical affection relatively more compared to other symptom clusters. The instrument that was used for the current study assessed avoidance, cognitive/emotional, and arousal symptoms using only a limited number of items which were combined to form one dimensional score. As such, we were not able to capture the differential relations between some clusters of posttraumatic stress symptoms and intimacy. Another potential explanation is that sexual intimacy is independent of PTSD symptoms and stress. Sexual intimacy encompasses more than mere sexual functioning. It entails being able to talk about each other’s sexual needs (Schaefer & Olson, 1981). As such, it is possible that victims are satisfied with how they communicate with their partner about sex while still experiencing relatively high PTSD and stress levels. Another explanation is that highly distressed victims ended their relationship in between waves. Therefore, it is possible they were not included in our follow-up assessments. A final reason for finding no association between psychological distress and sexual intimacy is that our sample exists of many victims with only moderate levels of psychological distress. Maybe this level of distress is not sufficient to affect victims’ intimacy ratings in future waves.

The second conclusion that can be drawn from our results is that there is evidence for a *reverse spillover effect*, especially for emotional intimacy. Emotional intimacy predicts future decreases in depression, anxiety, stress, and posttraumatic stress symptoms. This is consistent with earlier research among a variety of trauma victims showing that healthy relationship functioning might act as a buffer against future posttraumatic stress symptoms (Blais et al., 2021; Fredman et al., 2010; Levin et al., 2017; Zerach et al. 2019). These results suggest that being able to express emotions and thoughts to a partner and feeling understood by that romantic partner might act as a protective factor against later psychological distress following sexual violence (López-Zerón & Blow, 2015).

In a similar way, sexual intimacy predicts future decreases in later depression and anxiety scores. This suggests that being able to share sexual experiences and express mutual sexual needs is associated with increased individual well-being with respect to the victims’ mood and feelings of safety. Although being able to connect sexually seems to have beneficial effects on some domains of victims’ well-being, this does not account for all indicators of psychological distress. As such, sexual intimacy does not predict any changes in future stress and posttraumatic stress levels. It is possible that being able to communicate about sexual likes and dislikes means that couples try to avoid triggering the victim’s posttraumatic stress symptoms by altering the way they experience sexual intimacy. It is possible that posttraumatic stress symptoms then remain unchanged as the victim does not get the chance to associate certain forms of sexual intimacy with something other than the assault (Orlando & Koss, 1983). Another explanation might be that victims engage in complacency sex (i.e., sex to pleasure the partner; O’Callaghan et al., 2019). They might experience fewer depressive symptoms and anxiety because of feeling connected to their partner. However, this also might not alter the negative association of sexual activity with the assault (Orlando & Koss, 1983). Specifically, for stress, an additional explanation might be that stress is heavily influenced by multiple environmental factors (e.g., work-related stress, stress due to childcare, financial stress) and sexual intimacy alone might be insufficient to affect future fluctuations in stress.

Interestingly, whether the victim had disclosed the assault to their partner did not affect either victims’ psychological distress or victims’ intimacy ratings. To explain the absence of this association we can draw from the sexual violence disclosure literature. For example, the intimate partner’s reaction towards disclosure and how this is perceived by the victim might be more important than the disclosure itself. Studies on social reactions towards disclosure suggest that reactions that are perceived as supportive by victims might act as a protective factor against future psychopathology, whereas unsupportive reactions increase the risk of future psychopathology (Dworkin et al., 2019). It is therefore possible that the victims in our sample who disclosed to their partner received reactions they consider as being supportive.

### Practical Implications

Our study results suggest that in addition to individual trauma-focused treatment, victims and their partners might benefit from couples therapy. Couples therapy might aid in supporting couples to improve or maintain their emotional intimacy levels since these have shown to affect the victim’s individual functioning. Improving intimacy levels can be achieved by inviting the partner to therapy in vivo. This can be a possibility if the partner is informed about the sexual trauma and the victim agrees to their involvement in therapy. To improve emotional and sexual intimacy in couples, clinicians can support both partners in communicating their emotions and needs, listening to each other’s desires and trying to understand each other’s perspective (Kardan-Souraki, 2015).

In addition, couples therapy can help both partners in dealing with the aftermath of sexual violence including the victim’s psychological distress. Psychoeducation on the impact of a traumatic event like sexual violence and the reciprocal relation between individual distress and couple functioning should be provided (see for instance cognitive behavioral-couples therapy for PTSD; Monson et al., 2012). This information can increase understanding within the couple which might facilitate engaging in behaviors that improve intimacy within the couple. When victims did not disclose to their partner or when they do not agree on involving their partner in therapy, the clinician can consider including the partner’s perspective when working on intimacy-related topics by explaining the victim how distress can affect relationship intimacy and vice versa. In addition, together with the victim, the clinician can look for ways to increase intimacy levels within the relationship. As such, they can indirectly try to work on the couple’s relationship.

Another important consideration to discuss with the victim as a clinician is whether they should disclose the assault towards their partner. Although our study did not find evidence for the role of disclosure, it remains significant to offer support to victims in making decisions about sharing their experiences with their partners. Furthermore, it is vital to provide assistance to victims if they choose to disclose and to help both individuals in the couple to process the information together.

### Limitations and Suggestions for Future Research

Although our research is innovative in bidirectionally investigating psychological distress and relationship functioning in victims using a longitudinal design, some important limitations must be considered. First, despite the fact we started with a fairly large sample of victims in a romantic relationship there was a considerable drop-out over time. Only about one fourth of our initial sample participated in one or two future survey waves. This drop-out potentially increases the likelihood of underfitted models due to low power. However, we found multiple effects and as such, assume that no power issues were involved in our study. Drop-out analyses showed that higher stress levels and lower emotional and sexual intimacy levels during the first wave were associated with a higher likelihood of drop-out during the following waves. This means that the respondents in our longitudinal analyses had higher levels of individual and relational well-being. Second, it is possible that our community sample did not report extensive assault impacts, leading to some null effects. Additionally, the diversity in ecological factors affecting the assault impact might have influenced our results. Future studies should assess both clinical and community samples and take into account ecological factors that might affect the impact of the assault. Third, our counterintuitive findings with regard to posttraumatic stress symptoms might be due to the fact that our measurement of posttraumatic stress was not extensive enough. It did not differentiate between symptom clusters and as such, some expected associations might have been missed. Future research can benefit from using a more comprehensive assessment of posttraumatic stress symptoms. Fourth, the current study only assessed whether the assault happened within the past year. Since most changes in psychological distress occur during the first year following the assault (Stockman et al., 2023), it is possible that time since the assault might interact with victims’ psychological distress and relationship functioning. Future studies should include a more detailed measure of time since the assault as a covariate. Additionally, prospective studies starting from the moment victims experienced an assault with several follow-up assessment periods would be highly informative to investigate the bidirectional associations between psychological distress functioning and couple functioning following sexual violence. Fifth, our study only included the perspective of the victim on relationship intimacy following an assault. The partner’s perspective on how an assault affects the couple’s intimacy is also important. As such, a fruitful area for further research would be to include a dyadic assessment when investigating psychological distress and intimacy following sexual violence. Sixth, the current study assessed adjustment at one-year intervals, which only provides a longer-term snapshot of the bidirectional association between psychological distress and couple functioning. However, equally important is examining how short-term (e.g., using a daily diary) variations in psychological distress and couple intimacy might affect each other since this would improve the external validity of the current research findings. Seventh, the current study would be enhanced when using a more elaborated measurement of the assault disclosure to the intimate partner including how the partner reacted and how the victim perceived this reaction. Finally, our study did not track whether survivors broke up or initiated any new relationships in between waves. In addition, it might be interesting to evaluate potential differences in the associations of distress and intimacy between couples who were in a relationship prior to the assault and those who initiated the relationship post assault.

## Conclusion

The current study sought to examine whether sexual violence victims’ psychological distress was longitudinally and bidirectionally associated with victims’ intimacy in their romantic relationship. Generally, evidence for effects of victims’ psychological distress on their emotional and sexual intimacy and effects of intimacy on psychological distress were found. The results suggest that targeting intimacy levels within relationships might improve victims’ individual functioning and vice versa. Future research should additionally include dyadic assessments and more frequent assessments to enhance our understanding of the associations between individual functioning and couple functioning following sexual violence.

## Data Accessibility Statement

The datasets generated and/or analyzed during the current study are not publicly available due to their sensitivity. They are available from the corresponding author on reasonable request.

## Additional File

The additional file for this article can be found as follows:

10.5334/pb.1240.s1 Supplementary Tables. Supplementary table A: Survey overview for each cohort and wave. Supplementary table B: Wave 1 correlations between indicators of psychological distress, relational intimacy and participation in future waves. Supplementary table C: Solution for fixed effects of the final models for emotional and sexual intimacy.
