# Supplementary material for: Psychological Distress and Relational Intimacy following Sexual Violence: A Longitudinal Study
Source: Psychol Belg. 2024 Dec 17;64(1):185–200. doi: 10.5334/pb.1240 (PMC11661156; doi:10.5334/pb.1240)
Supplement: Supplementary Tables. — Supplementary table A: Survey overview for each cohort and wave. Supplementary table B: Wave 1 correlations between indicators of psychological distress, relational intimacy and participation in future waves. Supplementary table C: Solution for fixed effects of the final models for emotional and sexual intimacy. [file pb-64-1-1240-s1.pdf]

Supplementary Table A

Survey overview for each cohort and wave

	Nov 2019 – March 2020	Nov 2020 - March 2021	Nov 2021 - March 2022
Cohort 1	Wave 1<br>- Demographics<br>- DASS<br>- PAIR – Emotional and sexual intimacy<br>- THS – child abuse items<br>- Sexual violence since age 16<br>- Past year sexual violence<br>- Disclosure of sexual violence<br>- PTSD screener<br>- Identification code	Wave 2<br>- Identification code<br>- Demographics<br>- DASS<br>- PAIR – Emotional and sexual intimacy<br>- PTSD screener<br>- Past year sexual violence<br>- Disclosure of sexual violence	Wave 3<br>- Identification code<br>- Demographics<br>- DASS<br>- PAIR – Emotional and sexual intimacy<br>- PTSD screener<br>- Past year sexual violence<br>- Disclosure of sexual violence
Cohort 2		Wave 1<br>- Demographics<br>- DASS<br>- PAIR – Emotional and sexual intimacy<br>- THS – child abuse items<br>- Sexual violence since age 16<br>- Past year sexual violence<br>- Disclosure of sexual violence<br>- PTSD screener<br>- Identification code	Wave 3<br>- Identification code<br>- Demographics<br>- DASS<br>- PAIR – Emotional and sexual intimacy<br>- PTSD screener<br>- Past year sexual violence<br>- Disclosure of sexual violence

Note. DASS = Depression Anxiety Stress Scales; PAIR = Personal Assessment of Intimacy in Relationships; THS = Trauma History Screen; PTSD = Posttraumatic Stress Disorder

Supplementary Table B

Wave 1 correlations between indicators of psychological distress, relational intimacy and participation in future waves

	2.	3.	4.	5.	6.	7.
1. Posttraumatic stress	.473**	.457**	.406**	-.225**	-.142**	-.010
2. Depression		.721**	.744**	-.269**	-.154**	-.080
3. Anxiety			.767**	-.130**	-.088	-.078
4. Stress				-.163**	-.056	-.102*
5. Emotional intimacy					.585**	.111*
6. Sexual intimacy						.152**
7. Participation in future wave(s)						

Note. ** $p<.001$; * $p<.05$

Supplementary Table C

Solution for fixed effects of the final models for emotional and sexual intimacy

	Emotional intimacy				Sexual intimacy			
**Effect**	**Estimate**	**95% C.I. (Wald)**		**P (LRT)**	**Estimate**	**95% C.I. (Wald)**		**P (LRT)**
**Intercept**	**29.23**	**24.01**	**34.46**	**<.001**	**30.89**	**25.94**	**35.84**	**<.001**
**Wave (Time)**	-0.41	-1.24	0.41	.395	-0.09	-0.84	0.66	.198
**Cohort**	0.08	-1.03	1.19	.330	-0.95	-2.05	0.15	.450
**Age**	-0.08	-0.15	-0.01	**<.001**	**-0.12**	**-0.20**	**-0.05**	**<.001**
**Gender** (ref. Male)				.867				**.005**
Female	0.65	-2.26	3.56		0.46	-2.25	3.18	
Transgender	0.65	-2.80	4.09		-2.41	-5.64	0.83	
**Sexual orientation** (ref. Heterosexual)				.135				.476
LGB+	-0.41	-1.59	0.76		-0.08	-1.22	1.06	
**Relationship duration (in years)**	**-0.26**	**-0.37**	**-0.14**	**<.001**	**-0.19**	**-0.30**	**-0.08**	**<.001**
**Education** (ref. No higher)				.077				.910
Higher	0.67	-0.44	1.78		-0.41	-1.48	0.66	
**Employment** (ref. Working)				**.034**				.106
Student	-0.51	-1.92	0.91		-0.15	-1.52	1.22	
(Temporarily) not working	-1.27	-2.89	0.34		-0.65	-2.20	0.90	
**Child abuse** (ref. No)				.313				.115
Yes	0.57	-0.57	1.72		0.20	-0.91	1.30	
**Hands-off SV in past 12m** (ref. No)				.063				.399
Yes	-1.29	-3.04	0.46		-0.63	-2.24	0.99	
**Hands-on SV in past 12m** (ref. No)				.562				.417
Yes	0.77	-0.62	2.17		0.79	-0.54	2.13	
**Disclosure of SV to partner** (ref. No)				.092				.081
Yes	0.92	-0.34	2.19		0.92	-0.30	2.14	
**Disclosure of SV to formal support provider** (ref. No)				.309				.425
Yes	1.45	0.34	2.57		0.10	-0.96	1.16	
**Depression**	-0.04	-0.20	0.11	**<.001**	-0.05	-0.20	0.10	**<.001**
**Anxiety**	-0.02	-0.19	0.15	.074	**-0.20**	**-0.36**	**-0.03**	**.003**
**Stress**	**-0.20**	**-0.38**	**-0.02**	**.046**	-0.03	-0.20	0.14	.794
**Posttraumatic stress**	**-0.41**	**-0.70**	**-0.13**	**.004**	-0.13	-0.40	0.15	.365

Note. C.I. : Confidence Interval; LRT = Likelihood Ratio Test; LGB+ = self-identified as lesbian, gay, bisexual, pan-/omnisexual, asexual, or other; SV = Sexual Violence; 12m = 12 months.

Supplementary Table D

Solution for fixed effects of the final models for depression, anxiety, stress, and posttraumatic stress

	Depression				Anxiety				Stress				Posttraumatic stress			
**Effect**	**Estimate**	**95% C.I. (Wald)**		**P (LRT)**	**Estimate**	**95% C.I. (Wald)**		**P (LRT)**	**Estimate**	**95% C.I. (Wald)**		**P (LRT)**	**Estimate**	**95% C.I. (Wald)**		**P (LRT)**
**Intercept**	**12.51**	**6.35**	**18.68**	**<.001**	**16.43**	**10.72**	**22.14**	**<.001**	**15.46**	**9.89**	**21.02**	**<.001**	**4.81**	**2.28**	**7.35**	**<.001**
**Wave (Time)**	-0.46	-1.24	0.32	.065	-0.46	-1.06	0.15	**.009**	-0.38	-1.03	0.27	**.012**	**-0.96**	**-1.30**	**-0.61**	**<.001**
**Cohort**	0.52	-0.61	1.65	.241	0.00	-1.15	1.14	.454	0.31	-0.77	1.40	.230	-0.01	-0.46	0.44	.958
**Age**	-0.12	-0.19	-0.04	.482	**-0.14**	**-0.22**	**-0.07**	**.005**	**-0.12**	**-0.19**	**-0.05**	**.004**	0.01	-0.02	0.04	**.030**
**Gender** (ref. Male)				.348				.675				.441				.368
Female	1.81	-1.08	4.69		0.29	-2.64	3.21		2.23	-0.48	4.94		0.21	-1.00	1.42	
Trans	0.90	-2.55	4.35		-0.76	-4.17	2.64		0.39	-2.82	3.61		0.16	-1.27	1.59	
**Sexual orientation** (ref. Heterosexual)				**<.001**				**.001**				**<.001**				.133
LGB+	**2.00**	**0.83**	**3.16**		**1.44**	**0.29**	**2.58**		**1.45**	**0.35**	**2.55**		0.13	-0.34	0.60	
**Relationship duration (in years)**	0.08	-0.04	0.19	**<.001**	-0.05	-0.16	0.06	.187	-0.06	-0.16	0.05	.203	-0.03	-0.08	0.01	.330
**Education** (ref. No higher)				.465				**.003**				.121				.250
Higher	0.38	-0.73	1.49		-0.84	-1.94	0.26		-0.08	-1.13	0.97		0.10	-0.35	0.55	
**Employment** (ref. Working)				**<.001**				**<.001**				**.004**				**.001**
Student	0.72	-0.70	2.15		-0.36	-1.71	0.98		-0.37	-1.68	0.95		0.33	-0.24	0.91	
(Temporarily) not working	**2.65**	**1.05**	**4.25**		**1.83**	**0.31**	**3.35**		1.17	-0.32	2.66		0.40	-0.26	1.05	
**Child abuse** (ref. No)				**<.001**				**<.001**				**<.001**				**<.001**
Yes	**2.16**	**1.07**	**3.26**		**2.30**	**1.18**	**3.42**		**2.24**	**1.18**	**3.30**		**1.07**	**0.62**	**1.51**	
**Hands-off SV in past 12m** (ref. No)				.433				.127				.232				.304
Yes	0.23	-1.52	1.99		0.85	-0.49	2.19		0.60	-0.77	1.97		0.25	-0.54	1.03	
**Hands-on SV in past 12m** (ref. No)				.443				.663				.240				.562
Yes	0.76	-0.64	2.15		0.64	-0.57	1.84		1.00	-0.24	2.25		0.30	-0.27	0.87	
**Disclosure of SV to partner** (ref. No)				.325				.466				.581				.284
Yes	-0.38	-1.66	0.89		-0.29	-1.51	0.92		-0.18	-1.36	0.99		0.27	-0.24	0.79	
**Disclosure of SV to formal support provider** (ref. No)				.071				**.024**				**.017**				**<.001**
Yes	1.01	-0.05	2.07		**1.18**	**0.12**	**2.25**		**1.31**	**0.29**	**2.32**		**1.47**	**1.04**	**1.90**	
**Emotional intimacy**	**-0.18**	**-0.30**	**-0.06**	**<.001**	**-0.15**	**-0.26**	**-0.04**	**<.001**	**-0.21**	**-0.32**	**-0.10**	**<.001**	**-0.09**	**-0.14**	**-0.04**	**<.001**
**Sexual intimacy**	**-0.13**	**-0.26**	**-0.01**	**.040**	**-0.16**	**-0.28**	**-0.04**	**.007**	-0.09	-0.21	0.03	.129	-0.01	-0.06	0.04	.585

Note. C.I. : Confidence Interval; LRT = Likelihood Ratio Test; LGB+ = self-identified as lesbian, gay, bisexual, pan-/omnisexual, asexual, or other; SV = Sexual Violence; 12m = 12 months.
